# Impact of human-derived hemoglobin based oxygen vesicles as a machine perfusion solution for liver donation after cardiac death in a pig model

**DOI:** 10.1371/journal.pone.0226183

**Published:** 2019-12-11

**Authors:** Tatsuya Shonaka, Naoto Matsuno, Hiromichi Obara, Ryo Yoshikawa, Yuji Nishikawa, Yo Ishihara, Hiroki Bochimoto, Mikako Gochi, Masahide Otani, Hiroyuki Kanazawa, Hiroshi Azuma, Hiromi Sakai, Hiroyuki Furukawa

**Affiliations:** 1 Department of Surgery, Asahikawa Medical University, Asahikawa-shi, Hokkaido, Japan; 2 Department of Mechanical Engineering, Tokyo Metropolitan University, Hachioji-shi, Tokyo, Japan; 3 Department of Pathology, Asahikawa Medical University, Asahikawa-shi, Hokkaido, Japan; 4 Department of Cell Physiology, The Jikei University School of Medicine, Minato-ku, Tokyo, Japan; 5 Department of Pediatrics, Asahikawa Medical University, Asahikawa-shi, Hokkaido, Japan; 6 Department of Chemistry, Nara Medical University, Kashihara-shi, Nara, Japan; University of Cambridge, UNITED KINGDOM

## Abstract

The recent clinical application of perfusion technology for the machine preservation of donation after cardiac death (DCD) grafts has some advantages. Oxygenation has been proposed for the preservation of DCD liver grafts. The aim of this study is to clarify whether the use of HbV-containing preservation solution during the subnormothermic machine perfusion (SNMP) of the liver graft improves the graft function of DCD porcine livers in an *ex vivo* reperfusion model. Pig livers were excised after 60 minutes of warm ischemic time and were preserved under one of three preservation conditions for 4 hours. The preservation conditions were as follows: 4°C cold storage (CS group; N = 5), Hypothermic machine preservation (HMP) with UW gluconate solution (HMP group; N = 5), SNMP (21°C) with UW gluconate solution (SNMP group; N = 5), SNMP (21°C) with HbVs (Hb; 1.8 mg/dl) perfusate (SNMP+HbV group; N = 5). Autologous blood perfusion was performed for 2 hours in an isolated liver reperfusion model (IRM). The oxygen consumption of the SNMP and SNMP+HbV group was higher than the HMP groups (p < 0.05). During the reperfusion, the AST level in the SNMP+HbV group was lower than that in the CS, HMP and SNMP groups. The changes in pH after reperfusion was significantly lower in SNMP+HbV group than CS and HMP groups. The ultrastructural findings indicated that the mitochondria of the SNMP+HbV group was well maintained in comparison to the CS, HMP and SNMP groups. The SNMP+HbVs preservation solution protected against metabolic acidosis and preserved the liver function after reperfusion injury in the DCD liver.

## Introduction

Given the critical shortage of donor liver organs, the suitability of expanded criteria donors (ECDs) has been explored with increasing urgency. Consequently, placement on waiting lists carries a 10%–20% mortality rate [1]. However, the use of donated after cardiac death (DCD) liver grafts carries an increased risk of primary non-function or ischemia-reperfusion injury. Hypothermia has been accepted widely for preserving the transplanted organ. Cold storage (CS) has become the standard preservation technique in the clinical practice of liver transplantation due to its accessibility and low cost. However, CS has been shown to deteriorate the condition of DCD liver, resulting in severe reperfusion injury and graft dysfunction [2,3]. Furthermore, the cold ischemic time was shown to be a significant risk factors for ischemic-type biliary injury [4, 5].

Guarrera et al. showed that hypothermic (8–10°C) machine perfusion (HMP) improves the graft function and attenuates classical biochemical markers of liver preservation injury [6]. Other studies have reported that hypothermic oxygenated MP improves the viability and functional integrity of the liver graft, despite the lack of an oxygen carrier in the preservation perfusate [7, 8]. Dutkowski et al. reported that the application of hypothermic oxygenated perfusion appears to be well-tolerated, easy to use, and protective against early and later injuries [9]. These results indicate the importance of oxygenation during HMP. Various preservation temperatures except hypothermia during MP have been investigated in preclinical and clinical settings over the last 10 years to improve the outcomes and graft quality of DCD liver transplantation. However, normothermic MP (NMP; 37°C) has shown some advantages over HMP. Fondevila et al. reported that NMP further improves the function of DCD livers that were preserved for 90 min. after cardiac death [10]. However, NMP requires an oxygen carrier because the liver grafts need full metabolic support [11,12]. Alternatively, oxygenated subnormothermic MP (SNMP, 21°C) has been shown to reduce portal venous resistance and increase bile production, resulting in an increased hepatic functional capacity compared to HMP [13]. Furthermore, the oxygen requirements during SNMP are relatively low due to the reduced metabolic activity of the liver and increased oxygen consumption [14,15]. Many studies have shown that an oxygen carrier is needed under NMP, but few have demonstrated any oxygen carrier benefit under HMP and SNMP conditions.

We herein report the application of a new preservation solution wherein liver allografts are oxygenated under dual pressure in subnormothermic conditions with hemoglobin-based oxygen vesicles (HbV) as an oxygen carrier solution. In the present study, an isolated *ex vivo* reperfusion model (IRM) simulating reperfusion after transplantation using diluted autologous blood was evaluated to investigate its utility in MP for DCD livers. This procedure can reduce the need for animals in transplantation experiments. The aim of this study was to clarify whether or not the use of HbV-containing preservation solution during SNMP improves the liver graft function of DCD porcine livers in an *ex vivo* reperfusion model.

## Materials and methods

### Animals

Domestic female cross-bred Large-Yorkshire, Landrace, and Duroc pigs (approximately 25 kg, 2–3 months of age) were purchased from Daisetsusanrokusya Co., Ltd. Pigs were brought into the gauge 3–4 days before the experiment and fed 1.2–1.5 kg of food each day. The gauge was equipped with an automatic water supply system. The floor was made of wire mesh, and excrement was cleaned out twice a day. Starting at 12 h before the experiment, fasting management was performed in order to ensure the safe administration of general anesthesia. Excision procedures were conducted under inhalation anesthesia with isoflurane (Forane VR; Abbot Japan, Tokyo, Japan). All efforts were made to minimize suffering. After confirming that sedation had been sufficiently achieved, a midline abdominal incision was made, and the animal was prepared for liver exposing. The pig was injected KCL (10mEq) and leading to cardiac arrest. After 60 minutes, the liver was rapidly removed. The isolated liver is washed out with 500 ml of Eurocollins solution supplemented with glucose and 3000 units of heparin.

All animal experiments were conducted according to the Guide for the Care and Use of Laboratory Animals at Asahikawa Medical University. All animal studies and procedures were approved by Asahikawa Medical University Animal Research Committee (permit no. 14172).

### Perfusion preservation machine

**Fig 1:**
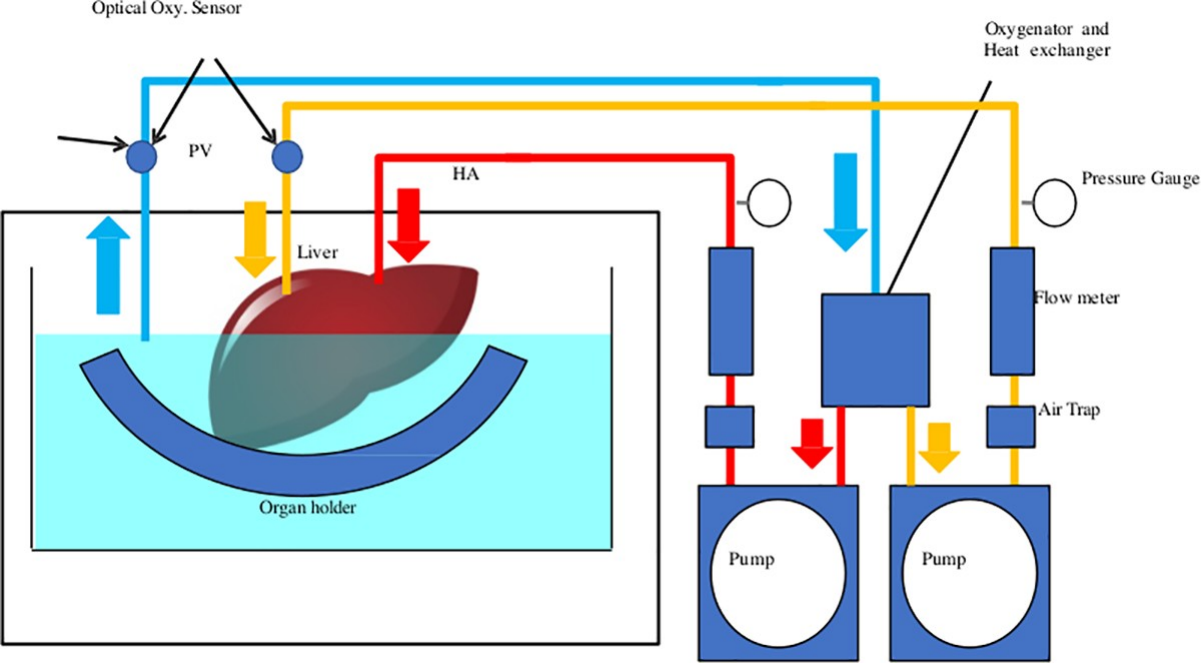
Machine perfusion system. This system consists of 2 separated perfusion circuits for the hepatic artery (HA) and portal vein (PV). Each circuit is equipped with a roller pump, a flow meter, a pressure sensor, and an air trap. An oxygenator was installed in the circuit for the PV and HA to measure the oxygen concentration of the perfusate. The temperature in the organ chamber was controlled by a heat exchanger and ice-cold water.

The grafts were perfused using an original MP system (Fig 1) [15]. This system consists of the hepatic artery (HA) and portal vein (PV) perfusion circuits. Each circuit consists of a roller-type pump (MasterFlex7520-40; Cole-Parmer, Bunker Court Vernon Hills, IL, USA), an electrical flow meter (VN05; Aichi Tokei, Japan for PV; FD-SS02, Keyence, Osaka, Japan for HA), a ceramic capacitance pressure sensor (KL76; Nagano Keiki, Nagano, Japan), and a custom-made air trap. An oxygenator (HP0-06 H-C; Senko Medical Instrument, Tokyo, Japan) was installed in the circuit for the PV and HA. Two optical oxygen sensors (NeoFox; Ocean Optics, Seattle, FL, USA) were installed to measure the oxygen concentration of the perfusate in the PV circuit. The temperature of the perfusate was controlled by a heat exchanger and ice-cold water. The flow rate of the PV was 0.22 mL/min/g (liver) and that of the HA was 0.06 mL/min/g (liver).

### Oxygenation

Oxygen was supplied to the liver grafts during MP (HMP and SNMP) using an oxygenator. The blending of oxygen and air was controlled with using the air blender of the oxygenator. The oxygen concentration was maintained to hold the minimum dissolved oxygen concentration for the calculation of the oxygen consumption at the outlet port.

### Experimental design

**Fig 2:**
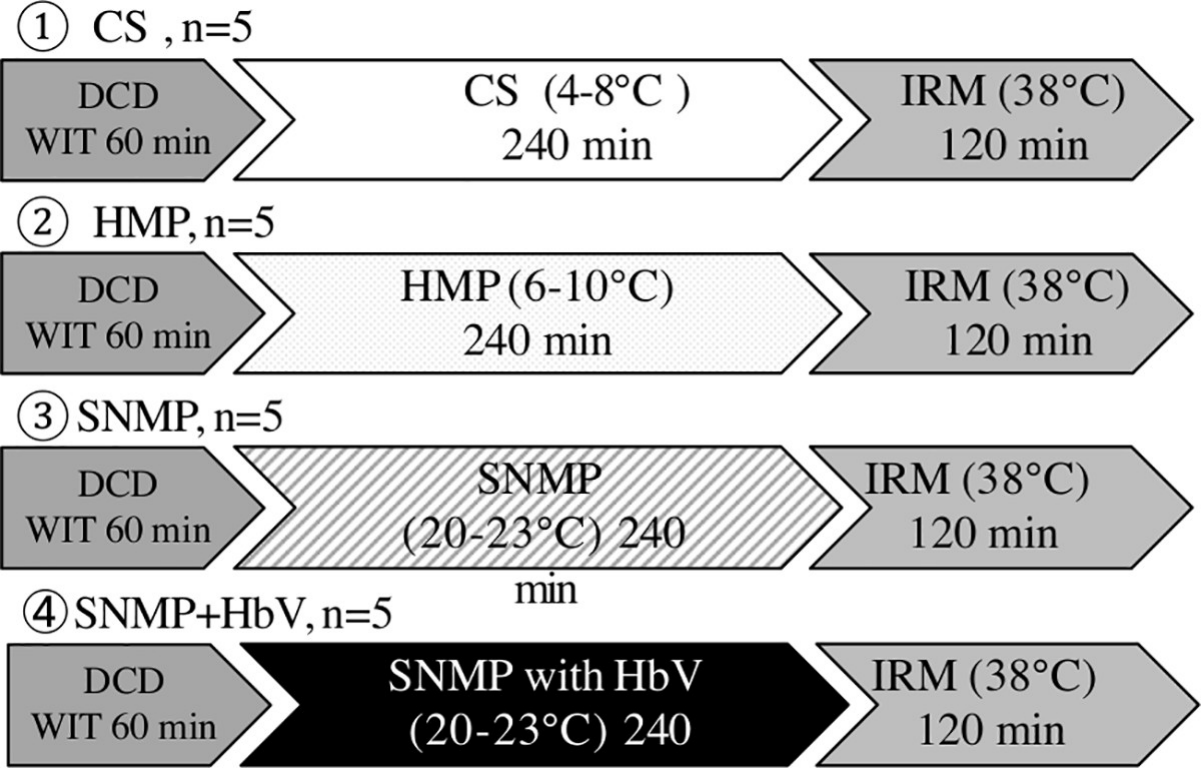
Experimental groups. Group 1: The livers were procured under a warm ischemia time of 60 min and preserved by CS (4–8°C, n = 5). Group 2: The livers were procured under warm ischemia time of 60 min and preserved by HMP (6–10°C, n = 5) with modified gluconate University of Wisconsin solution. Group 3: The livers were procured under warm ischemia time of 60 min and preserved SNMP with modified gluconate University of Wisconsin solution (22°C, n = 5). Group 4: The livers were procured under warm ischemia time of 60 min and preserved SNMP with HbVs containing preservation solution (22°C, n = 5).

Grafts were procured with a warm ischemia time (WIT) of 60 min. and were preserved under CS, HMP or SNMP with HbVs for 4 hours. The perfusate used a modified gluconate University of Wisconsin solution. We used four perfusion conditions in the present study (Fig 2):

1. CS group: liver grafts were preserved with CS for 4 h after 60 min of cardiac arrest. The CS group is cooled by ice. 5–8 degrees means the temperature of the liver which was cooled with ice (n = 5).
2. HMP group: liver grafts were perfused with HMP for 4 hours after a WIT of 60 min (n = 5).
3. SNMP group: liver grafts were perfused with SNMP for 4 hours after a WIT of 60 min (n = 5).
4. SNMP+HbV (SNMP with HbV) group: liver grafts were perfused with SNMP; HbVs for 4 hours after a WIT of 60 min (n = 5).

### Human-derived hemoglobin vesicles

Sakai et al. demonstrated human-derived hemoglobin vesicles (HbVs) as hemoglobin-derived oxygen carriers (HBOCs), which encapsulate a purified and concentrated hemoglobin solution in phospholipid vesicles, like red blood cells (RBCs) [16]. HbVs were prepared from out-of-date nucleic-acid amplification testing-inspected RBCs provided by the Japanese Red Cross. The characteristics of HbVs included no blood type, protection against infection due to pasteurization and nanofiltration, protection against the toxic effects of molecular hemoglobin, and stability and biocompatibility [17]. Human-derived HbVs were used as HBOCs. HbVs were developed 18–20 months after confirming their molecular stability [18].

### IRM

Yoshikawa et al. previously described an IRM [19]. The IRM can be useful for the evaluation of the utility of MP. Briefly, following preservation, all organs were rinsed with 500 mL of cold Euro-Collins solution for 20 min, and were subsequently exposed at room temperature for 10 min without perfusion to imitate the slow rewarming of the graft during surgical implantation *in vivo*. The CS group is perfused with the enough amount perfusate before reperfusion. The livers were then reperfused with oxygenated diluted autologous blood at 38°C. The autologous blood was diluted with 6000 ml of a solution containing saline (40%), low molecular dextran (20%), calcium (5%), sodium bicarbonate, heparin sodium, and potassium. The hematocrit was maintained at around 10–12%. The oxygenator was regulated to achieve the following physiological blood gas values: pO_2_, approximately 150–200 mmHg; pCO_2_, approximately 30–50 mmHg). HA perfusion was set at 80 mmHg and automatically maintained by a roller pump connected to a pressure sensor placed in the inflow line immediately before the arterial cannula. Perfusion of the PV was maintained under a constant flow rate of 0.8 mL/min/g.

### The viability assessment during MP

Aspartate aminotransferase (AST) and lactate dehydrogenase (LDH) were taken from the perfusate every 30 min and were analyzed to determine the viability of the preserved liver grafts using agents of standard clinical grade. The blood gas and lactate concentrations in perfusate were measured using the iStat program (Abbott, Cicago, NJ, USA).

To assess tissue damage, AST and LDH were analyzed. A blood gas analysis was performed to assess the electrolyte (Na, K, Ca and Cl), lactate and glucose concentrations, and acid/base physiology (pH, pO_2_, pCO_2_, HCO_3_ and base excess). Lactate was measured as a marker of metabolic disorders. The PV and hepatic artery pressure (HAP) was monitored.

### Histology

Tissue samples were collected to assess hepatocellular injury before, during and after preservation, post-reperfusion and at the necropsy performed at the end of the study. All liver samples were fixed in 10% buffered formalin, embedded in paraffin, sectioned (5 mm) and stained with hematoxylin and eosin for histological evaluation after preservation and reperfusion.

### Microscopic findings

Preparation of osmium macerated samples for observation by a scanning electron microscope was described previously [20]. In brief, a fixative mixture of 0.5% PFA and 0.5% glutaraldehyde in 0.1 MPB (pH 7.4) was macerated with liver samples for 30 min at 4°C. After fixation, the samples were directly immersed in 1% OsO_4_ in 0.1 MPB for 6 h at 4°C. Washed with 0.1 M PB and samples were cryoprotected with 25% and 50% dimethyl sulfoxide for 30 min. The specimen was frozen on a deeply cooled metal plate with liquid nitrogen. The frozen samples were cracked and transferred into 50% dimethyl sulfoxide for thawing. Samples were washed 3 times with 0.1 MPB and then transferred to 0.1% OsO4 diluted with 0.1 MPB for 90 h at 22°C and macerated with osmium. Samples were then immersed for 1 h in 1% OsO_4_ in 0.1 M PB, rinsed with 0.1 M PB, and conductively stained with 1% OsO_4_ in 1% tannic acid and 0.1 M PB. The samples were then dehydrated in ascending concentrations of ethanol (70%, 80%, 90%, 95%, and 100%) and transferred into 2-Methyl-2-propanol. The samples were frozen and dried in a freeze-dryer (ES-2030; Hitachi Koki Co., Ltd., Tokyo, Japan). After drying, samples were placed on an aluminum metal plate and then coated with platinum-palladium in an ion-sputtering apparatus (E1010; manufactured by Hitachi Koki). The specimens were then examined by field emission scanning electron microscopy (SEM) (S-4100; Hitachi High Technologies).

### Statistical analyses

The results are presented as the mean ± standard deviation (SD). *T*-tests were performed using the Microsoft Excel 2013 software program (Microsoft, Seattle, WA, USA). After proving the assumption of normality, differences between groups were tested using a *t*-test. A repeated measures analysis of variance was performed using the R software program (version 3.1.2) and the EZR software program [21]. P values of <0.05 were considered to indicate statistical significance.

## Results

### 1. HAP and oxygen consumption during perfusion preservation. (Figs 3 and 4)

In the HMP group, the HA pressure after 120 minutes and 240 minutes of perfusion preservation was 38.4 ± 5.4 mmHg and 34.9 ± 4.4 mmHg, respectively. In the SNMP group, the HA pressure after 120 and 240 minutes of perfusion preservation was 29.4 ± 14.5 mmHg and 37.4 ± 17.1 mmHg, respectively. In the SNMP+HbV group, the HA pressure after 120 and 240 minutes of perfusion preservation was 20.0 ± 5.7 mmHg and 21.7 ± 7.3 mmHg, respectively. The HAP during perfusion tended to be lower in the HbV + SNMP group compared to the SNMP group, but there was no significant difference. In the HMP group, the oxygen consumption after 120 and 240 minutes of perfusion preservation was 16.7 ± 4.3mg/min/100g liver and 12.3 ± 2.9mg/min/100g liver, respectively. In the SNMP group, the oxygen consumption after 120 and 240 minutes of perfusion preservation was 40.7 ± 6.2mg/min/100g liver and 38.6 ± 4.8mg/min/100g liver, respectively. In the SNMP+HbV group, the oxygen consumption after 120 and 240 minutes of perfusion preservation was 50.3 ± 21.6 mg/min/100g liver and 47.9 ±2.4 ml/min/100g liver, respectively. In the SNMP and SNMP+HbV group, the amount of oxygen used was much higher than that of the HMP group. Oxygen consumption during perfusion tended to be higher in the HbV + SNMP group compared to the SNMP group, but there was no significant difference.

**Fig 3:**
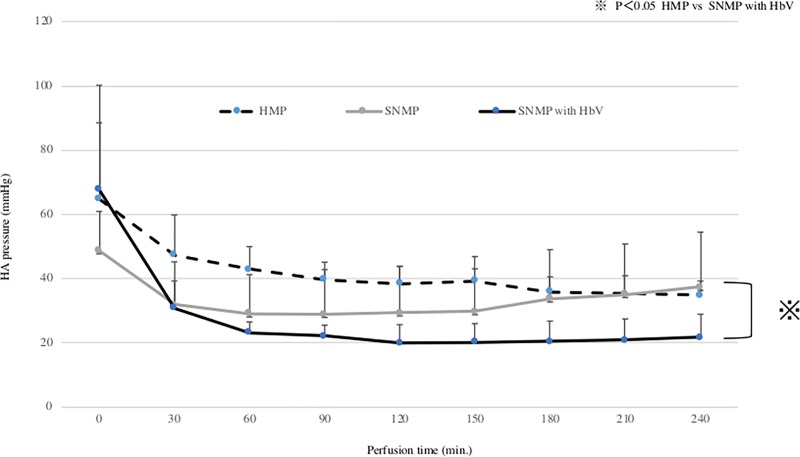
Hepatic artery pressure during machine preservation HA pressure (HAP) after each perfusion condition during machine preservation. The HAP was significantly higher than after SNMP+HbV. HMP vs. SNMP with HbV: ※ p<0.05.

**Fig 4:**
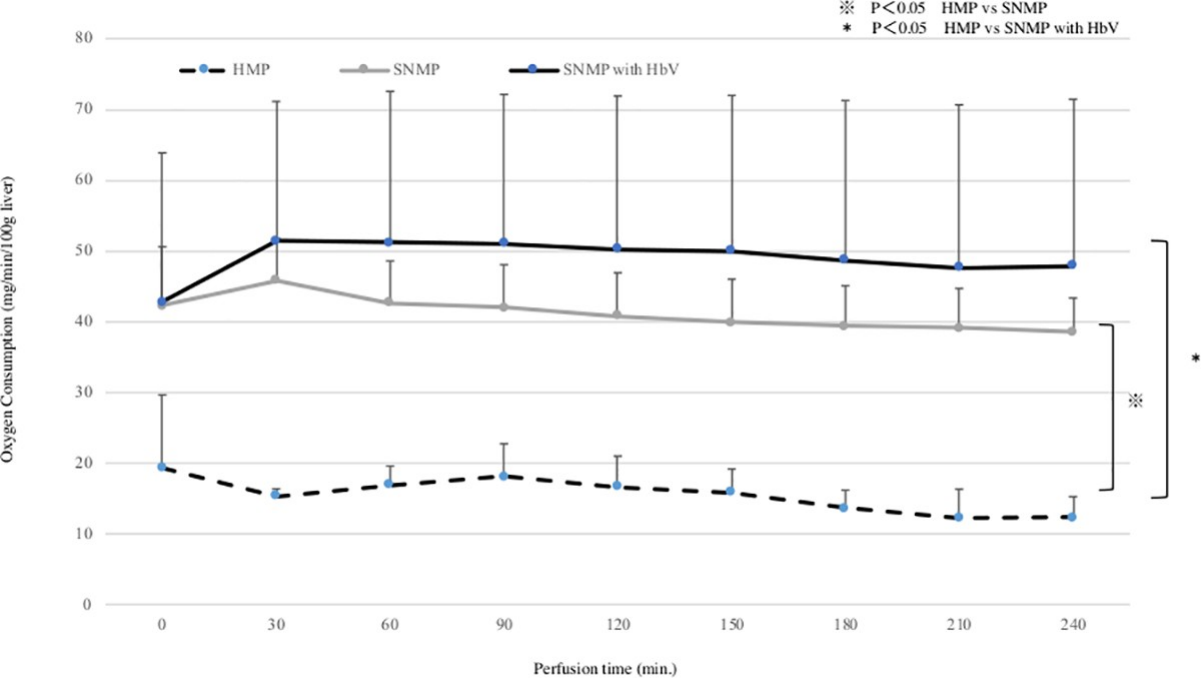
Oxygen consumption during machine preservation. The oxygen consumption in SNMP+HbV was significantly higher than after HMP. SNMP+HbV vs. HMP: *p<0.05, SNMP vs. HMP: ※ p<0.05.

### 2. AST and LDH in perfusion solution during perfusion preservation (Figs 5 and 6)

In the HMP group, the AST level after 120 and 240 minutes of perfusion preservation was 2020 ± 871 IU/L and 2204 ± 932 IU/L, respectively. In the SNMP group, the AST level after 120 and 240 minutes of perfusion preservation was 2072 ± 691 IU/L and 2149 ± 726 IU/L, respectively. The AST level of the SNMP group was almost the same as that of the HMP group. In the SNMP+HbV group, the AST level after 120 and 240 minutes of perfusion preservation was 1666 ± 1206 IU/L and 1650 ± 1105 IU/L, respectively, which was lower in comparison to the SNMP and HMP groups. In the HMP group, the LDH level after 120 and 240 minutes of perfusion preservation was 2830 ± 597 IU/L and 3100 ± 670 IU/L, respectively. In the SNMP group, the LDH level after 120 and 240 minutes of perfusion preservation was 2524 ± 928 IU/L and 2704 ± 958 IU/L, respectively. In the SNMP+HbV group, the LDH level after 120 and 240 minutes of perfusion preservation was 1861 ± 1327 IU/L and 2000 ± 1340 IU/L, respectively, which was lower than that of the SNMP and HMP group.

**Fig 5:**
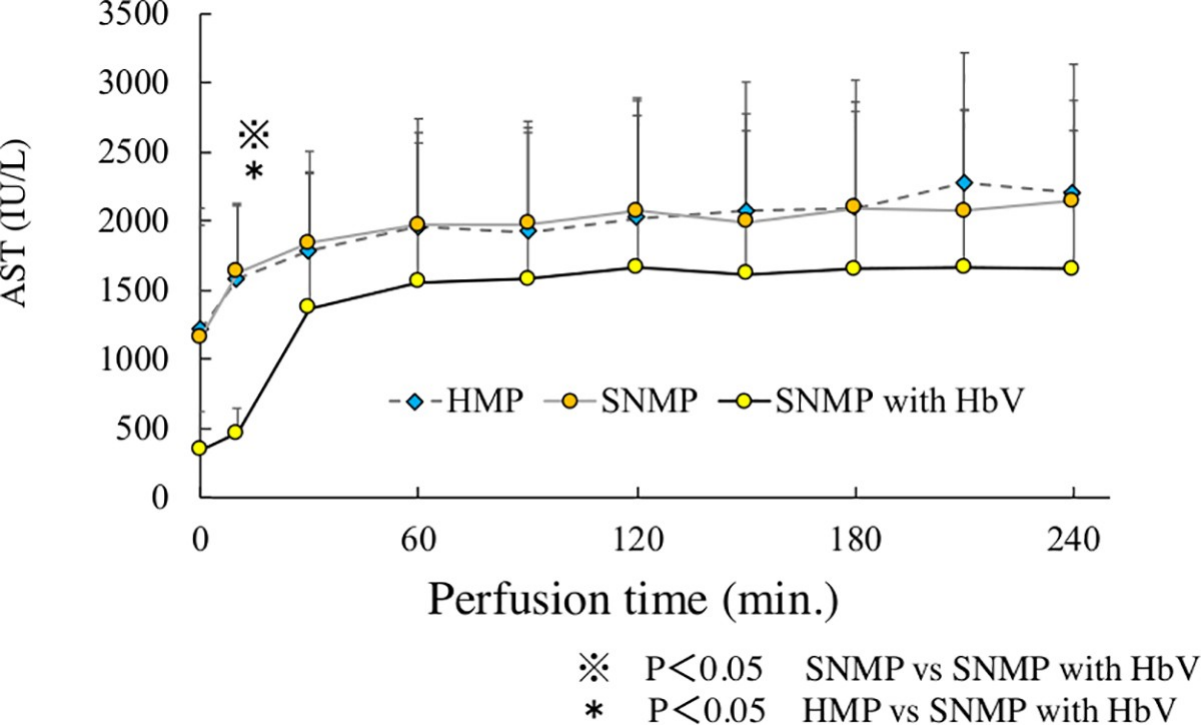
AST during machine preservation are shown. AST in SNMP with HbVs was lower than HMP and SNMP at every point after reperfusion.

**Fig 6:**
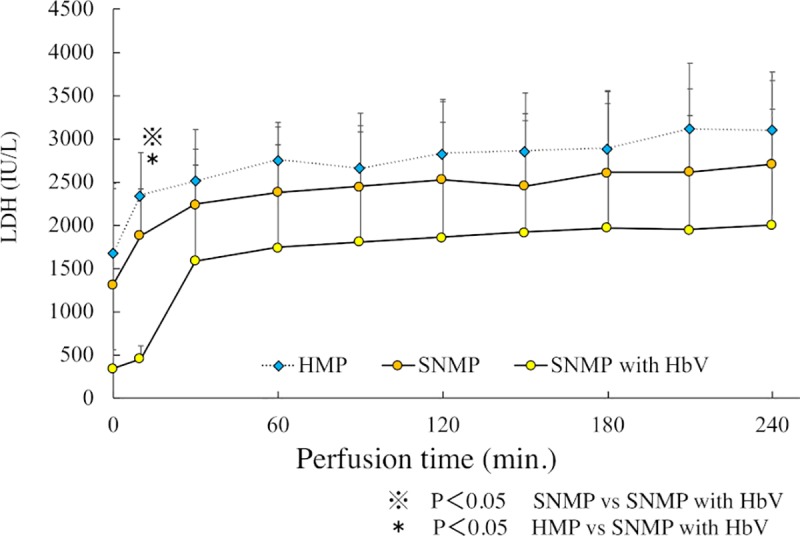
LDH during machine preservation are shown. LDH in SNMP with HbVs was lower than HMP and SNMP at every point after reperfusion.

### 3. Changes in PV and HA pressure (Figs 7 and 8)

The change of portal vein pressure was calculated using the following formula: (PV pressure after 60 minutes of reperfusion)—(PV pressure after 0 minutes of reperfusion) / PV pressure after 0 minutes of reperfusion. In the CS group, the change of PV pressure was 0.64 ± 0.66 which was the highest among the four groups. In the HMP group, the change of PV pressure was 0.21 ± 0.75 at 60 minutes of reperfusion. In the SNMP group, the change in PV pressure was 0.02 ± 0.20. The value in the SNMP with HbV group was -0.06 ± 0.24, which was lower than that of the CS group. These results showed that the change of PV pressure was lowest in the SNMP+HbV group. The change in hepatic artery pressure was calculated using the following formula: (HA pressure after 60 minutes of reperfusion)—(HA pressure after 0 minutes of reperfusion) / HA pressure after 0 minutes of reperfusion. In the CS group, the change of HA pressure was 0.87 ± 0.56 times. In the HMP group, the change of HA pressure was 1.82 ± 0.60, which was the highest among the four groups and was significantly higher than that of the HMP and SNMP+HbV groups. In the SNMP group, the change of HA pressure was 1.18 ± 0.59. In the SNMP+HbV group, the change of HAP was 0.46 ± 0.59. These results showed that the SNMP+HbV group was hemodynamically stable in the isolated reperfusion phase. But There was no significant difference between HbV + SNMP group and SNMP group of the changes of HA and PV.

**Fig 7:**
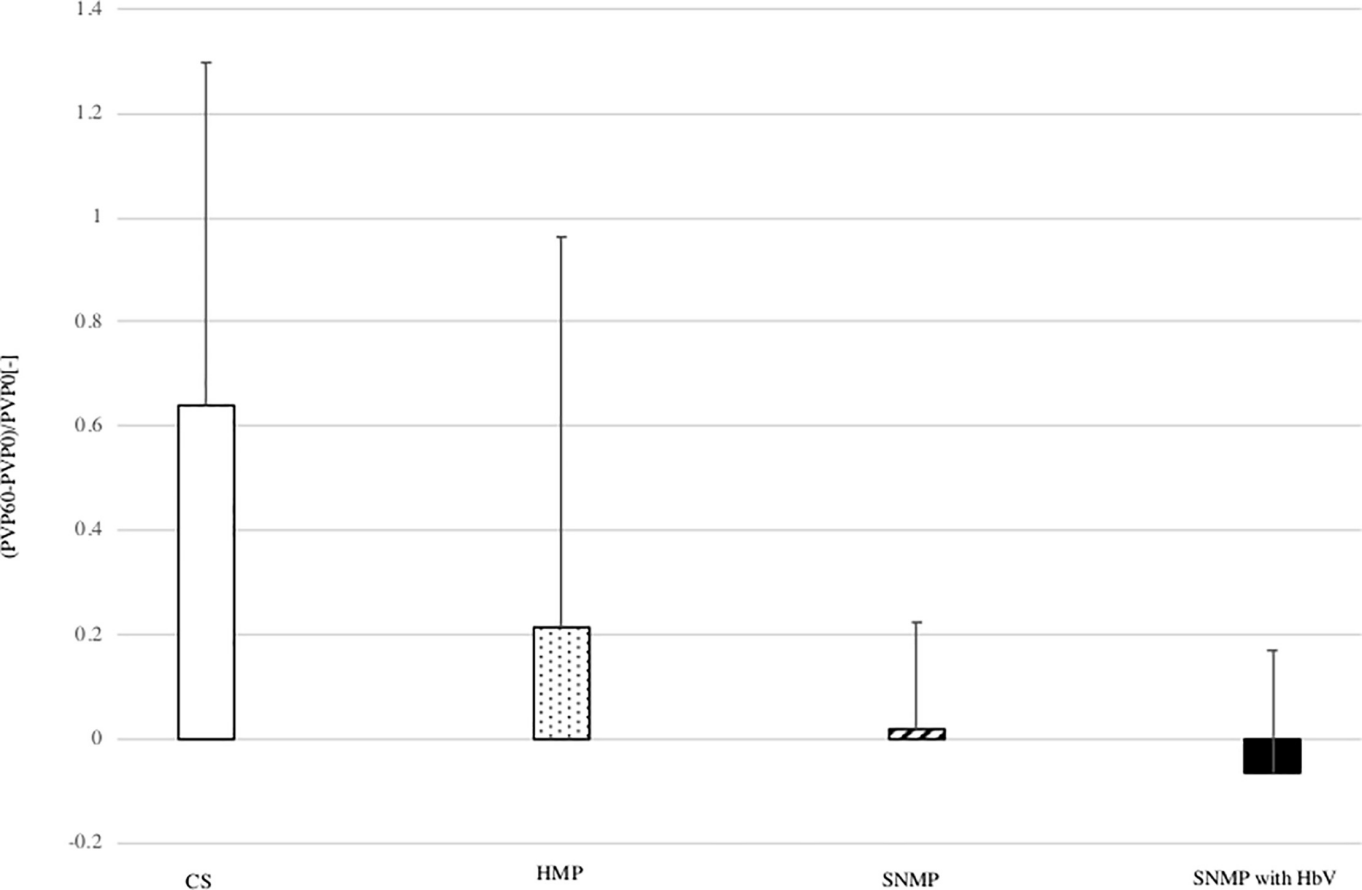
The change in the portal vein pressure. The change in the portal vein pressure was calculated as follows: (PV pressure after 60 minutes of reperfusion)—(PV pressure after 0 minutes of reperfusion) / PV pressure after 0 minutes of reperfusion. The change of PV pressure (PVP) after each perfusion condition during IRM are shown. The PVP after CS was higher than HMP and SNMP and SNMP with HbV.

**Fig 8:**
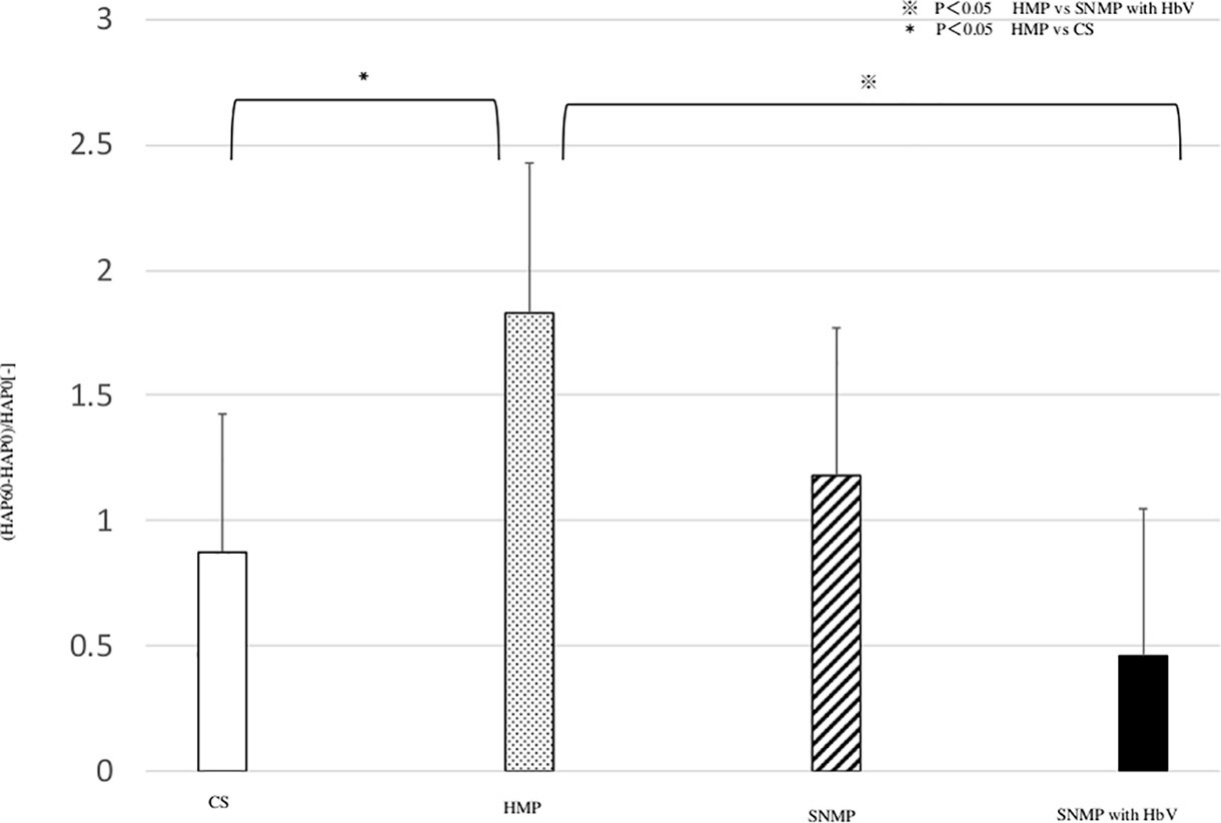
The change of hepatic artery pressure. The change of hepatic artery pressure was calculated as follows: (HA pressure after 60 minutes of reperfusion)—(HA pressure after 0 minutes reperfusion) / HA pressure after 0 minutes of reperfusion. The change of HA pressure (HAP) after each perfusion condition during IRM are shown. The HAP after HMP was higher than after CS and SNMP and SNMP with HbV. HMP vs. SNMP with HbV: ※ p<0.05, CS vs HMP: * p<0.05.

### 4. AST and LDH in the perfusion solution after reperfusion (Figs 9 and 10)

In the SNMP+HbV group, the AST was 1128 ± 175 IU/L after 120 minutes of reperfusion. The AST level of this group was the significantly lower in comparison to the other three groups. In the SNMP group, the AST level was 1408 ± 230 IU/L after 120 minutes of reperfusion, which was significantly lower than that of the CS group. In the HMP group, the AST level was 1599 ± 417 IU/L after 120 minutes of reperfusion, which was significantly lower than the level of the CS group. In the CS group, the AST level was 3636 ± 1316 IU/L after 120 minutes of reperfusion.

In the SNMP+HbV group, the LDH level was 1523 ± 350 IU/L after 120 minutes of reperfusion, which was significantly lower in comparison to the other three groups. In the SNMP group, the LDH level was 1604 ± 132 IU/L after 120 minutes of reperfusion, which was significantly lower in comparison to the CS group. In the HMP group, LDH was 1810 ± 288 IU/L in 120 minutes of reperfusion, which was significantly lower in comparison to the CS group.

**Fig 9:**
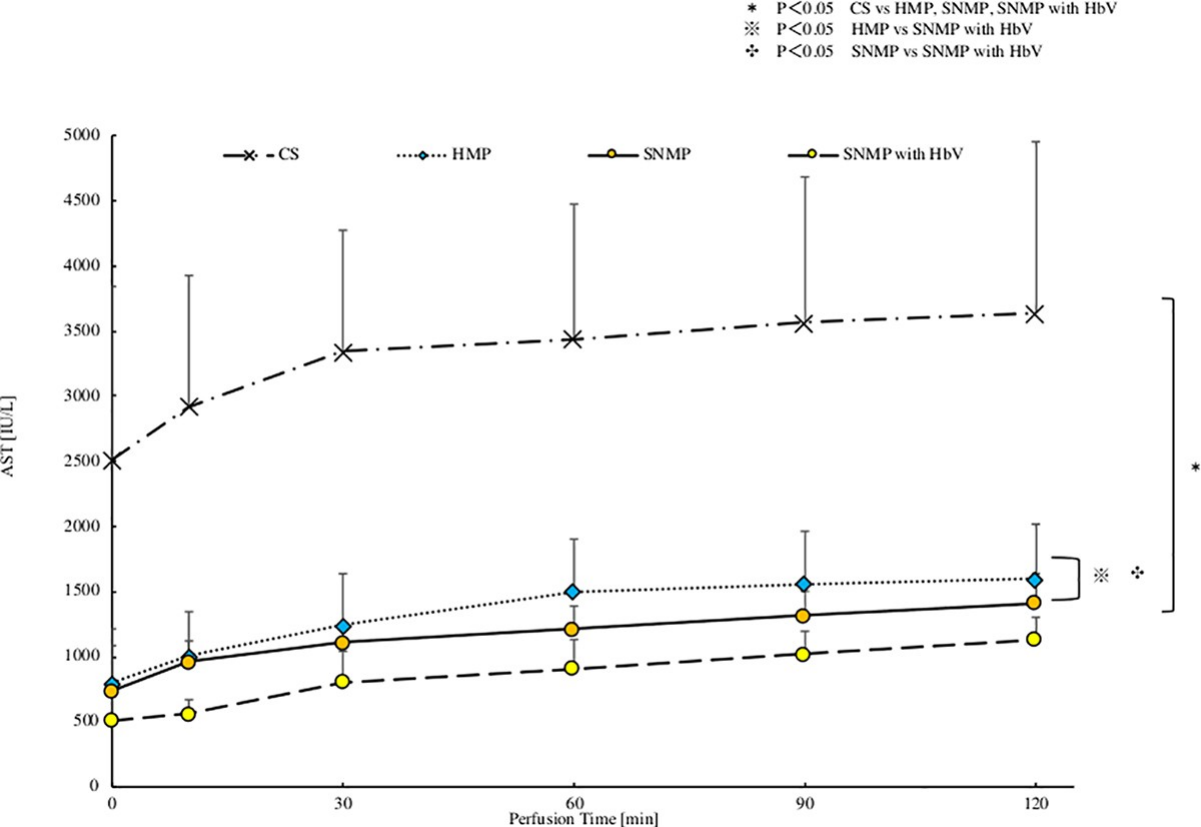
AST level after reperfusion. AST after each perfusion condition during IRM are shown. AST after CS was significantly higher than HMP, SNMP and SNMP with HbV groups. The AST level of the SNMP with HbV group was significantly lower in comparison to the other groups: *P<0.05 CS vs HMP, SNMP, SNMP with HbV, ※P<0.05 HMP vs SNMP with HbV, ✣P<0.05 SNMP vs SNMP with HbV.

**Fig 10:**
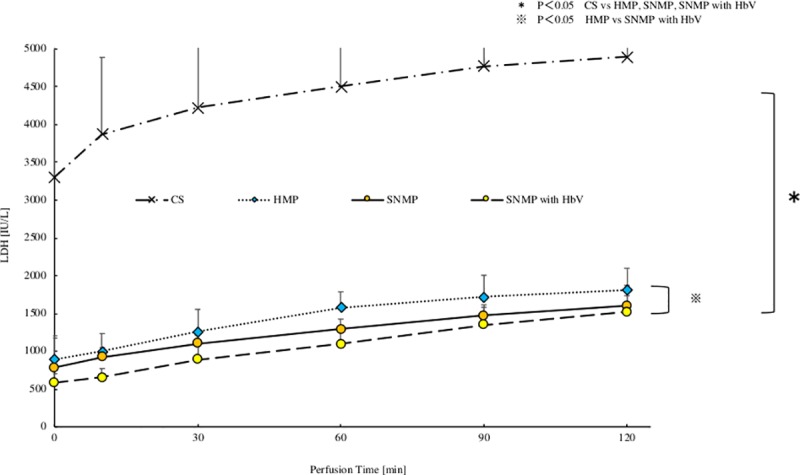
LDH after reperfusion. LDH after each perfusion condition during IRM are shown. LDH with IRM after CS was remarkably higher compared with HMP, SNMP, SNMP with HbV groups. The SNMP with HbV group showed a significantly lower LDH level in comparison to the CS and HMP group. *P<0.05 CS vs HMP, SNMP, SNMP with HbV, ※P<0.05 HMP vs SNMP with HbV.

### 5. pH and lactate after reperfusion (Figs 11 and 12)

The PH after 120 min. of reperfusion were 7.13 ± 0.08, 7.34 ± 0.2, 7.26 ± 0.14, 7.34 ± 0.12 (CS, HMP, SNMP, SNMP+HbV group). The decrease of PH was higher in the CS group (-0.24 ±0.10). The decrease of pH was -0.20 ± 0.08 during 120 minutes of reperfusion in the HMP group. In the SNMP group, the decrease in pH was -0.19 ± 0.07 after 120 minutes of reperfusion. In the SNMP+HbV group, the decrease of pH was -0.09 ± 0.17 during 120 minutes of reperfusion, which was the lowest among the four groups. In the SNMP with HbV group, the decrease of pH was significantly lower than that in the CS and HMP groups. In the CS group, the increase of lactate was 6.09 ± 0.65 mmol/L at 120 minutes of reperfusion, which was significantly higher than that in the HMP, SNMP, SNMP with HbV groups. The increase of lactate was2.61 ± 0.52 mmol/L in HMP and 1.59 ± 0.68 mmol/L in SNMP. On the other hands, in the SNMP+HbV group, the increase of lactate was 1.55 ± 2.97mmol/L after 120 minutes of reperfusion, which was the lowest among four groups. But There was no significant difference between HbV + SNMP group and SNMP group of lactate.

**Fig 11:**
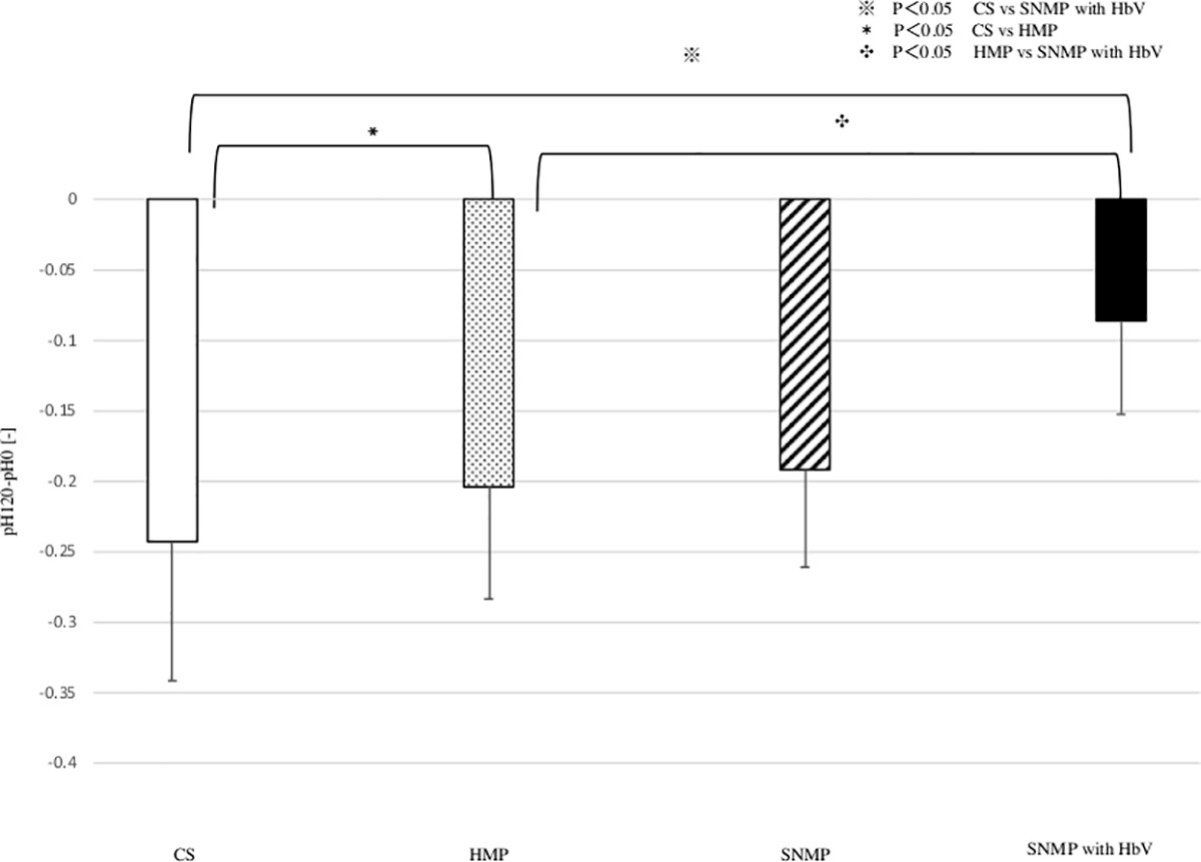
The changes of pH after reperfusion. pH after each perfusion condition during IRM are shown. pH with IRM after CS was remarkably higher compared with HMP and SNMP with HbV group. CS vs. HMP: * p<0.05, CS vs SNMP with HbV: ※ p<0.05, CS vs. HMP: ✤ p<0.05.

**Fig 12:**
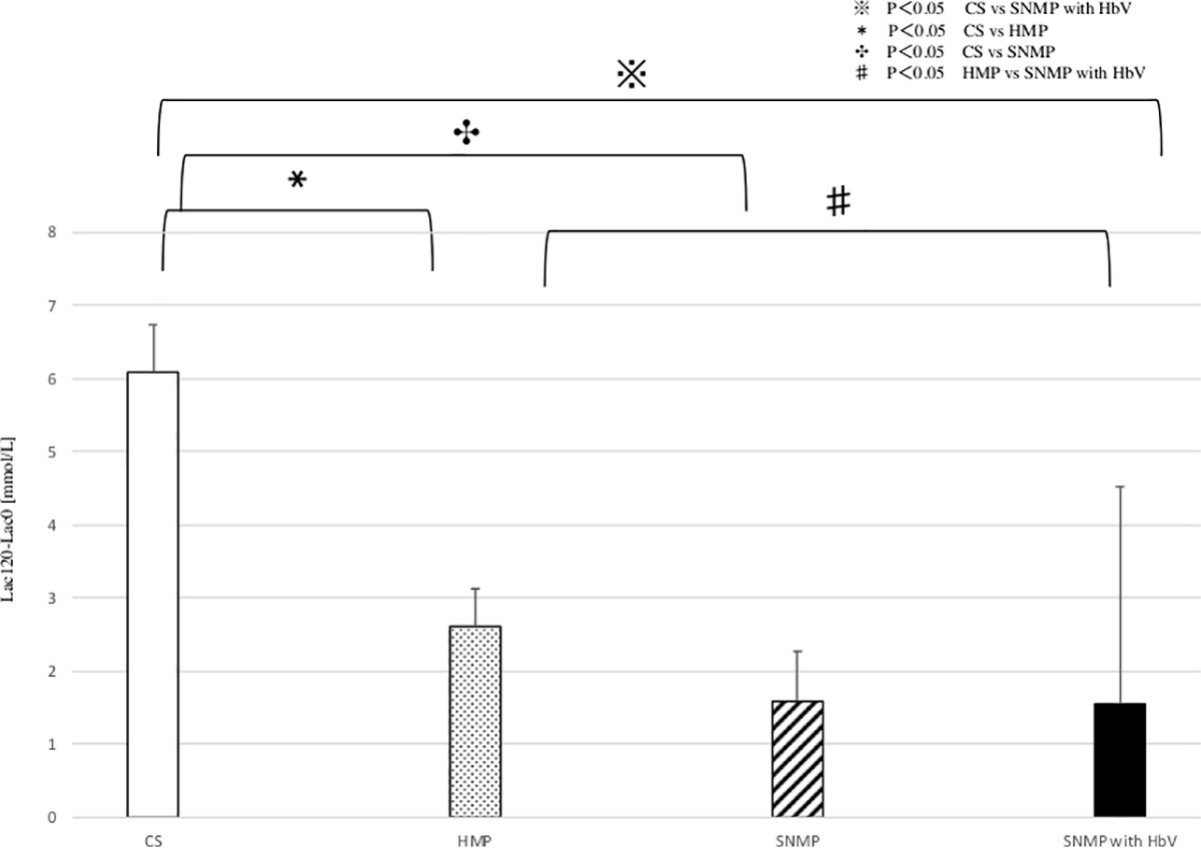
The changes of Lactate after reperfusion. The changes of lactate after CS was significantly higher than HMP and SNMP and SNMP with HbV groups. The changes of lactate in SNMP with HbV group was significantly lower than HMP group. CS vs HMP: * p<0.05 CS vs. SNMP: ✤ p<0.05, CS vs SNMP with HbV: ※ p<0.05, HMP vs SNMP with HbV: ♯ p<0.05.

### 6. Histology (Fig 13A–13l))

**Fig 13:**
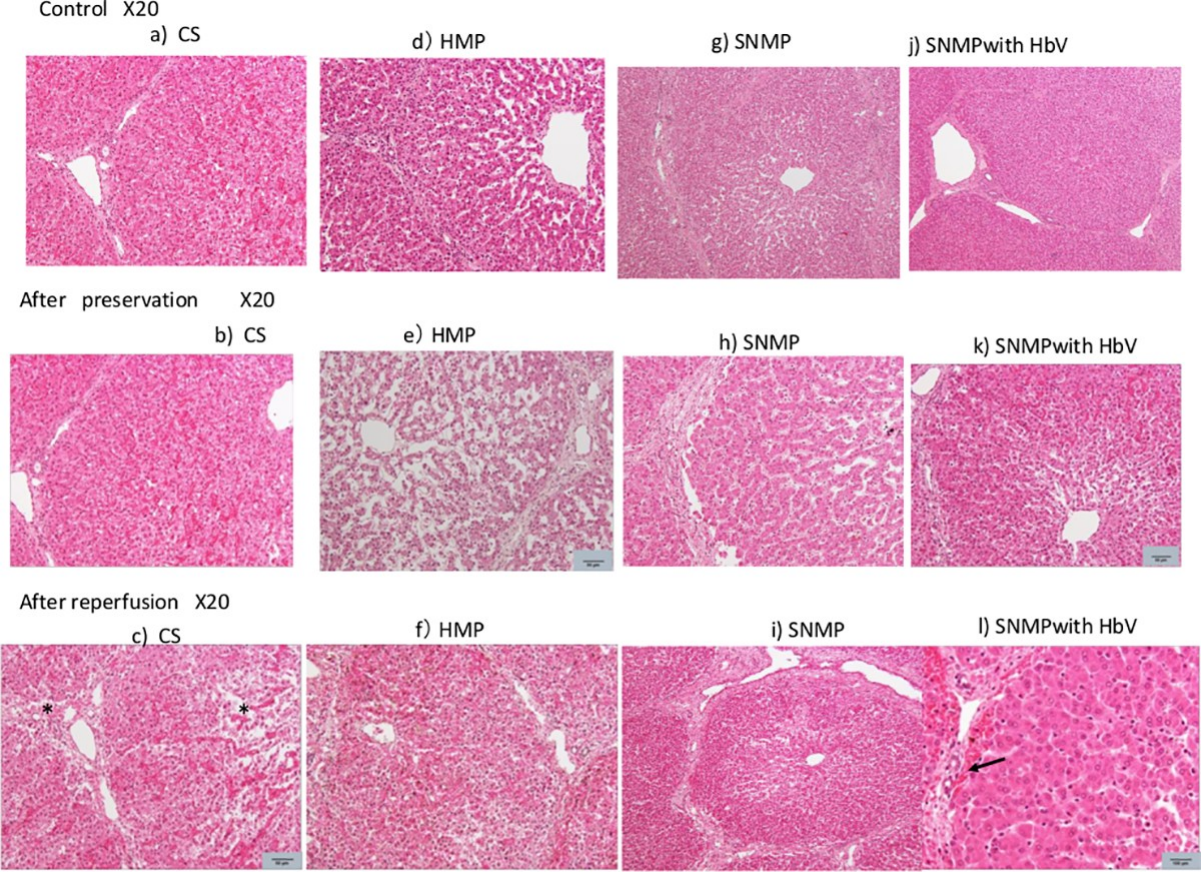
The pathological findings control (pre preservation), after 4hours of preservation and after 2 hours of reperfusion. a), b) and c) show the CS group at control (pre-preservation), the end of preservation and reperfusion; d), e) and f) show the HMP group; and g), h) and i) show the SNMP group; j), k) and l) show the SNMP with HbV group at each time point, respectively. The comparison of the CS, HMP and SNMP groups revealed that disorder around the central veins and bile duct damage were suppressed in the HbV+SNMP group (arrow). Edema around the sinusoid was worsened in the CS group (*). In the HbV+SNMP group, no HbVs remained at the end of reperfusion.

Fig 13 shows the pathological findings after control (pre preservation), 4hours of preservation and after 2 hours of reperfusion: a), b) and c) show the CS group at the end of preservation and reperfusion; d), e) and f) show the HMP group; and g), h) and i) show the SNMP group, j), k) and l) show SNMP with HbV groups at each time respectively. In the “Control” state, changes observed in lipid deposits of hepatocytes occurred, but there was no significant change in the state between the 4 groups. The comparison of the CS, HMP and SNMP groups revealed that disorder around the central vein damage were suppressed in the HbV+SNMP group. Edema around the sinusoid was worsened in the CS group. In the HbV+SNMP group, no HbVs remained at the end of reperfusion.

### 7. SEM (Fig 14A and 14B)

**Fig 14:**
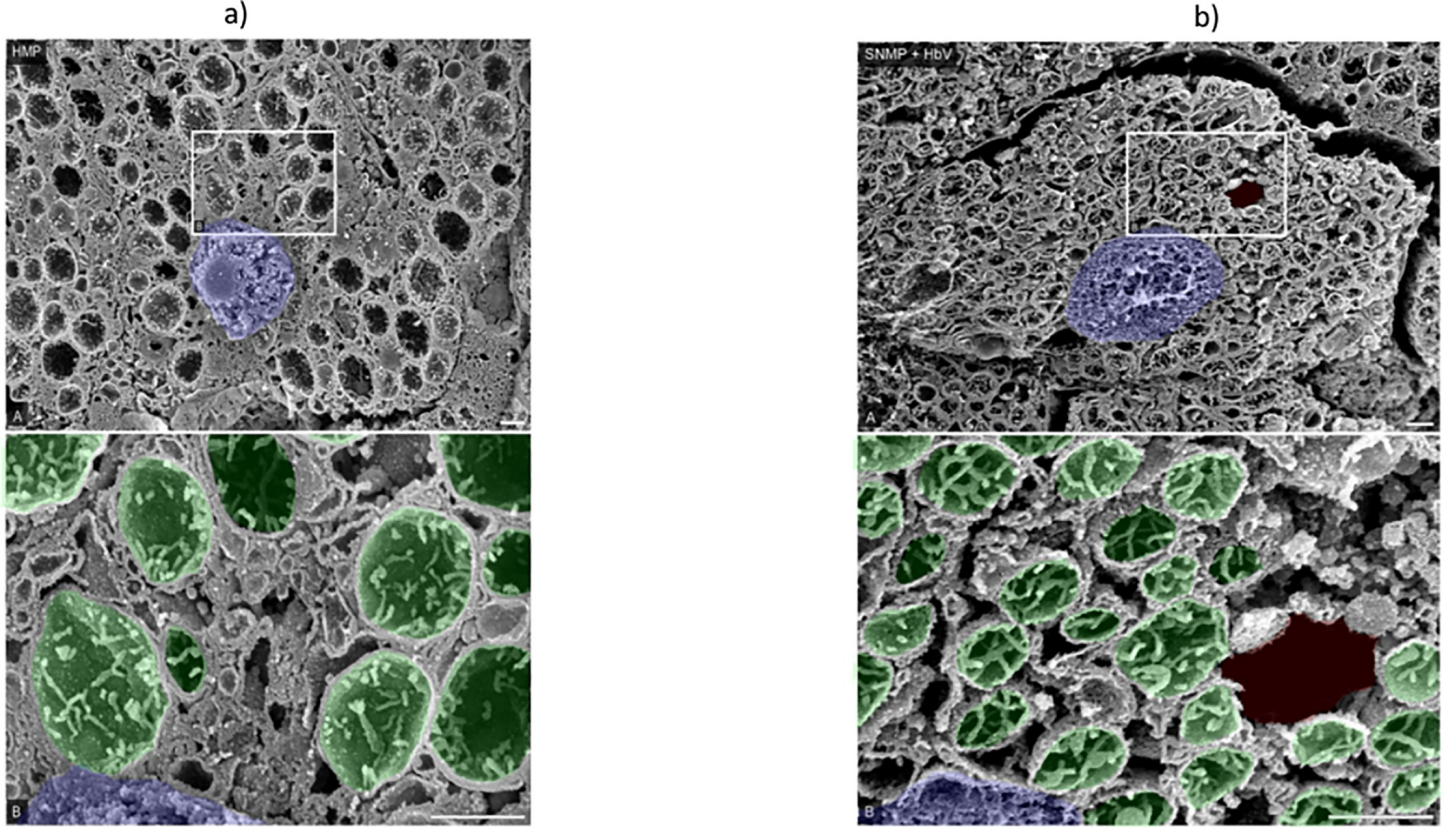
**(a) Observation by SEM.** Observation by SEM showed that the presence of mitochondria in the cytoplasm and reticulated endoplasmic reticulum in hepatocytes in HMP samples (a) Strongly swollen mitochondria as a reflection of their damage under the low oxygenated condition appeared in the hepatocytes (a). In contrast, the treatment of the SNMP with HbVs reduced the frequency of appearance of the swollen mitochondria. **(b) Observation by SEM.** High magnified observation showed that the mitochondria appeared to have a normal function as reflected to their oxygenated condition with HbV. Bars = 1 μm.

Observation by SEM showed that the form of nucleus and mitochondria in the cytoplasm in hepatocytes in HMP samples (Fig 14A) higher magnification, strongly swollen mitochondria as a reflection of their damage under the low oxygenated condition appeared in the hepatocytes (Fig 14A). These findings of the organelles in hepatocytes in HMP well correspond to our findings described previously^19^. In contrast, the treatment of the SNMP with HbVs reduced the frequency of appearance of the swollen mitochondria (Fig 14B). High magnified observation showed that the mitochondria appeared to have a normal function as reflected to their oxygenated condition with HbVs (Fig 14B). Bars = 1 μm.

## Discussion

MP technology has some advantages with regard to DCD liver grafts over CS[6, 12]. For example, oxygenated and transportable HMP systems have been shown to reduce the ischemic damage to DCD liver grafts compared with CS [22]. Oxygenated SNMP improves not only the oxygen uptake but also the adenosine triphosphate content and reduces the lactate levels of the donor liver [13,14]. Our previous study showed that the oxygen consumption was related to the graft temperature and associated with the effluent LDH level [23]. However, hypothermic conditions seem to have limited utility in graft preservation. In addition, cold-induced preservation injury can be caused by reactive oxygen species, leading to mitochondrial injury and lipid peroxidation [24,14]. MP with oxygenation improves the liver graft viability. Morito et al. demonstrated that the concentrations of effluent enzymes in SNMP were lower than those in HMP [23]. These results suggest that oxygen consumption with SNMP reduced the levels of effluent liver enzymes, thereby ameliorating liver graft injury. Tolboom et al. [25] also reported that SNMP recovered ischemic damaged rat livers after 1 h WIT. Furukori et al. [26] reported that SNMP preservation with rewarming maintained the AST and LDH levels in the effluent. However, these findings had limitations regarding the prediction of general perfusion conditions—in particular the conditions of optimized oxygenated perfusion solutions.

The use of whole-blood *ex vivo* reperfusion as a pig model for transplantation, was associated with reduced bile duct injury in DCD grafts in comparison to CS livers [27]. In recent years, there have been some reports on the use of oxygen carriers for perfusion of the donor liver. Fontes et al. [28] performed liver transplantation after SNMP in perfusion with Hemopure® in a pig model. Hemopure® was reported to improve the oxygen carrying ability 8-fold, and the graft function of the liver that was perfused with Hemopure® was improved. That study further showed that, at 21°C, the perfusate with Hemopure® kept methemoglobin levels low, indicating that perfusate with Hemopure® (hemoglobin = 3.5g/l) provided reliable oxygen delivery and effective CO_2_ removal. The additional advantages of the hemoglobin oxygen carrier (HBOC) had unlimited availability, a long storage period, and low risk of disease dissemination [29, 30].

In the present study, we investigated the influence of SNMP+HbVs oxygen carrier using a pig isolated liver reperfusion model. We used HbVs produced using human-hemoglobin. HbVs are a hemoglobin oxygen carrier (HBOC) created using expired RBCs [16], and they have demonstrated stability, encapsulation efficiency, and biocompatibility [17]. The characteristics of HbVs include no blood type and a long storage life. HbVs are 250 nm in size, which means they cannot pass through the liver sinusoid.

This study showed that SNMP with oxygen carrier could reduce reperfusion injury in the DCD donor liver. As shown in Figs 3 and 4, superior preservation conditions were observed in the SNMP+HbV group. Obara et al; reported that DCD liver grafts require excessive levels of oxygen [25]. Oxygen was effectively used by the DCD liver grafts in SNMP+HbV, as shown in Fig 4 and it cannot effectively consume oxygen without an oxygen carrier.

Perfusate with HbVs was found to transport oxygen, even under low Ht conditions. Obara et al. reported that HA pressure during MP was a reliable marker when using DCD liver grafts [31]. As shown in Figs 7 and 8, the change of HA pressure and PV pressure in the SNMP+HbV group were maintained at lower levels than in the HMP and SNMP groups. The concentrations of liver enzymes in effluent during preservation were lower than those in the HMP and SNMP group. As a result, SNMP+HbVs produced preconditioning effects and protected against liver dysfunction after reperfusion.

The present study also showed that SNMP with HbVs decreased the liver enzyme levels (AST, LDH) during the reperfusion phase (Figs 9 and 10). Liu Q et al. [32] also reported that perfusate containing an oxygen carrier was effective in normothermic machine perfusion. We thought that performing perfusion with SNMP increased the oxygen supply to the liver tissue, leading to organ protection and contributing to a reduction in the liver enzyme levels.

These results indicate that rapid rewarming following hypothermia at the time of reperfusion, even after 4 h of ischemic time, resulted in preservation-induced damage to the hepatocytes of the DCD liver and was associated with severe reperfusion injury. SNMP itself, may be effective for reducing the rates of reperfusion injury and shear stress. However, SNMP without any oxygen carrier cannot support metabolic disorders. This study demonstrated that the pH and lactate level after reperfusion remained maintained low in the SNMP+HbV group, as shown in Figs 11 and 12.

The storage of energy in mitochondria during preservation is also important. Bochimoto et al. [20] described the appearance of abnormal vacuoles and invagination of mitochondria in hepatocytes after 1 h warm ischemia by SEM. In the present study, the hepatocytes preserved by HMP showed extremely swollen mitochondria, while the SNMP+HbV groups had hepatocytes with healthy-looking mitochondria, abundant vacuoles, and membranous structures sequestrating cellular organelles. As shown in Fig 14, the mitochondria size was recovered and well-maintained in the SNMP+HbV group compared with the HMP group. Furthermore, the ultrastructure in the hepatocytes of liver grafts changed depending on the temperature conditions and presence of an oxygen carrier. In this study, the ultrastructural findings, as shown in Fig 14, revealed that mitochondria size was recovered and well maintained in SNMP+HbV group than HMP. Further studies are needed to investigate the mechanisms through which SNMP+HbV perfusion protects the bile duct blood supply and protects against injury after transplantation. In addition, the protective effect was limited because of the short period of observation after reperfusion.

Finally, we examine the validity of Hct in this isolated liver reperfusion model. Maione F et al. [33] review the perfusate composition of the model during reperfusion. Since the perfusate causes ischemia-reperfusion injury, whole blood should be used in reperfusion phase. This review demonstrated that the Hct in the experiment is about 20%, but there are some that are higher or lower than that. Yoshikawa et al. [19,34] showed the effectiveness of our evaluation system. Qiang Liu et al[35].showed the potential role for objective evaluation of reperfusion. The value of Hct10-12% in our model is a sufficiently acceptable.

This study was associated with some limitations. It was an experimental pig model, and transplantation was not performed. Furthermore, the results are not sufficient for predicting the effects of MP and HbVs in a living body, including the immune system effects.

In conclusion, SNMP+HbVs solution based on UW could alleviate hepatocellular reperfusion injury in the donor liver. Oxygenated SNMP+HbVs appears to be promising preservation method that may improve the functional recovery of the DCD liver during reperfusion.

## Supporting information

S1 Dataset The raw data in this study. (XLSX)
